# Primary submucosal nodular plasmacytoma of the stomach: a poorly recognized variant of gastric lymphoma

**DOI:** 10.1186/1746-1596-8-30

**Published:** 2013-02-20

**Authors:** Maki Kanzawa, Chihoko Hirai, Yukiko Morinaga, Fumi Kawakami, Shigeo Hara, Hiroshi Matsuoka, Tomoo Itoh

**Affiliations:** 1 Department of Diagnostic Pathology, Kobe University Hospital, Hyogo, Japan; 2 Department of Diagnostic Pathology, Osaka Medical Center, Osaka, Japan; 3 Department of internal medicine, division of medical oncology/hematology, Kobe University Hospital, Hyogo, Japan

**Keywords:** Plasmacytoma, Solitary, Stomach, Submucosa, Lymphoma

## Abstract

**Abstract:**

Gastric plasmacytoma (GP) is a rare variant of gastric lymphomas. In the exceptional event that a patient presents with GP, the lesion occupies the mucosal layer in the vast majority of cases. Here we report a case of nodular plasmacytoma confined to the submucosa with no evidence of *Helicobacter pylori* (Hp) infection. The patient was a 59-year old female presenting with no particular symptoms. The tumor was well-demarcated and consisted of a diffuse monomorphic proliferation of plasma cells with numerous lymphoid follicles scattered throughout the tumor. The mucosal surface was intact and not associated with any tumor nodules. The cells were diffusely positive for CD79a, Bob1, EMA and IgA and consistently negative for CD3, CD19, CD20, PAX5, CD56, IgM and IgG. Additionally, *in situ* hybridization demonstrated clonality in the form of λ light-chain restriction. This submucosal nodular proliferation pattern of plasmacytoma is poorly recognized and considered to be a novel variant of lymphoma.

**Virtual Slides:**

The virtual slide(s) for this article can be found here: http://www.diagnosticpathology.diagnomx.eu/vs/3489998708673079

## Background

Extranodal marginal zone lymphomas of mucosal associated lymphoid tissue (MALT lymphoma) and diffuse large B cell lymphomas are well-documented as the most common subtypes of GI lymphomas [1]. Within this context it is also important to note that MALT lymphoma is frequently associated with *Helicobacter pylori* (Hp) infection. In addition to featuring a large population of lymphocytes forming distinct lymphoepithelial lesions, about a third of MALT lymphoma cases will present with varying degrees of plasmacytic differentiation [2]. Immunoproliferative small intestinal disease (IPSID) in particular, which is a subtype of MALT lymphoma and primarily observed in the Middle East, typically shows striking plasmacytic differentiation [2].

On the opposite end of the spectrum, extraosseous plasmacytoma (EP), which is characterized by a localized and monoclonal expansion of plasma cells in tissues other than bone, arise in the upper respiratory tract which includes the oropharynx, nasopharynx, sinuses and larynx [2]. The next most frequent site of mass lesion occurrence is the stomach; however, this is extremely rare and accounts for less than 5% of all EPs [3]. Furthermore, the vast majority of conventional gastric plasmacytomas have been documented primarily in the mucosal layer [4-6].

This report details an exceptionally rare case of a nodular plasmacytoma occupying the gastric submucosa without an underlying Hp infection. To the best of our knowledge, only two similar cases have been reported to date and thus this condition can be effectively defined as a poorly recognized entity of primary gastric lymphoma.

## Case presentation

### Clinical history

**Figure 1:**
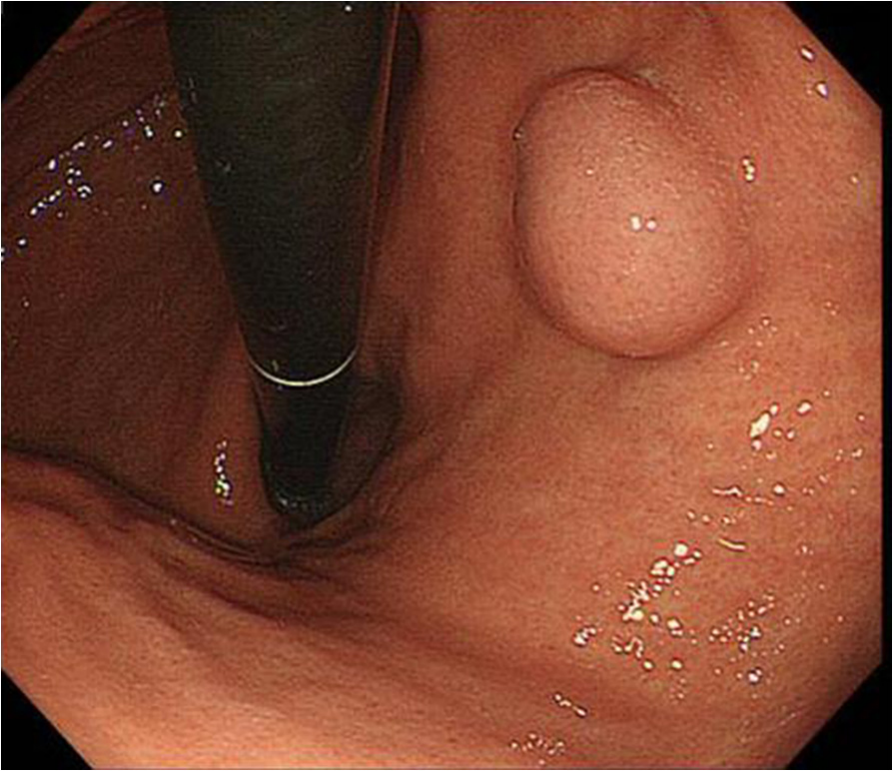
**Esophagogastroduodenoscopy.** Well-demarcated, elevated lesions on the posterior wall of the gastric corpus.

A 59-year-old female with a past medical history of thyroid papillary carcinoma was referred to our hospital due to a gastric tumor identified during a mass screening esophagogastroduodenoscopic (EGD) examination, which revealed a smoothly elevated lesion, 2 cm in diameter, occupying the posterior walls of the gastric corpus (Figure 1). No ulcerative change was observed on the mucosal surface, the patient was free of any accompanying symptoms and laboratory study findings were all within normal limits. Based on a diagnosis of the submucosal tumor not otherwise specified, a partial gastrectomy was performed.

Features of multiple myeloma were not apparent and the patient’s serum calcium, hemoglobin and creatinine levels were within normal limits. No significant findings on a skeletal X-ray examination were noted and positron emission tomography/computed tomography (PET/CT) showed no other lesions. Finally, serum and urine protein electrophoresis yielded normal results. No additional treatment was performed. The present study was approved by the Ethics Committee at Kobe University Graduate School of Medicine.

### Gross features

The surface mucosa of the tumor was smooth and normal-looking. The resected tumor measured 2.0×1.5×1.5 cm, was well demarcated, and consisted of a whitish and elastic soft mass located in the submucosal layer. The tumor had a homogenous, tan-colored cut surface and was focally hemorrhagic.

### Microscopic features

**Figure 2:**
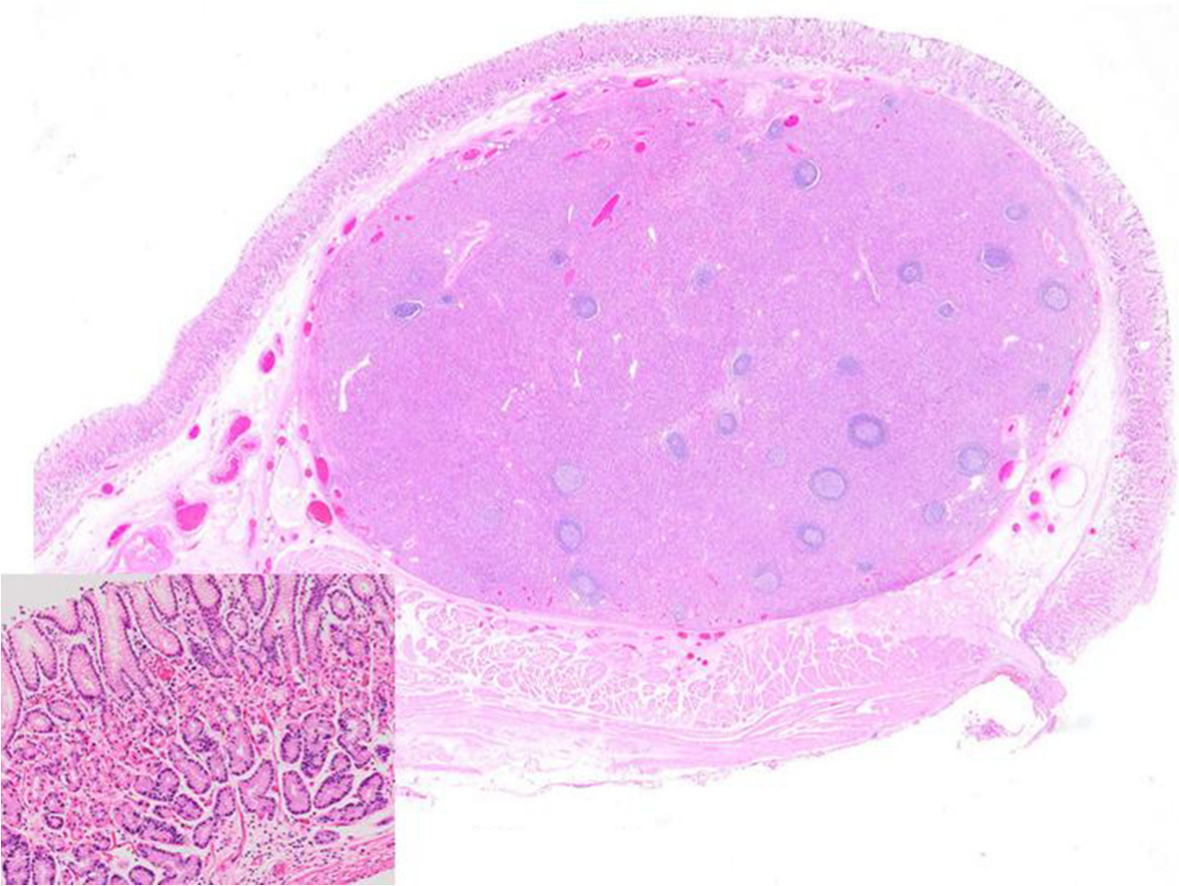
**The tumor forms a submucosal gastric nodule (HE stain).** Inset shows normal mucosa.

**Figure 3:**
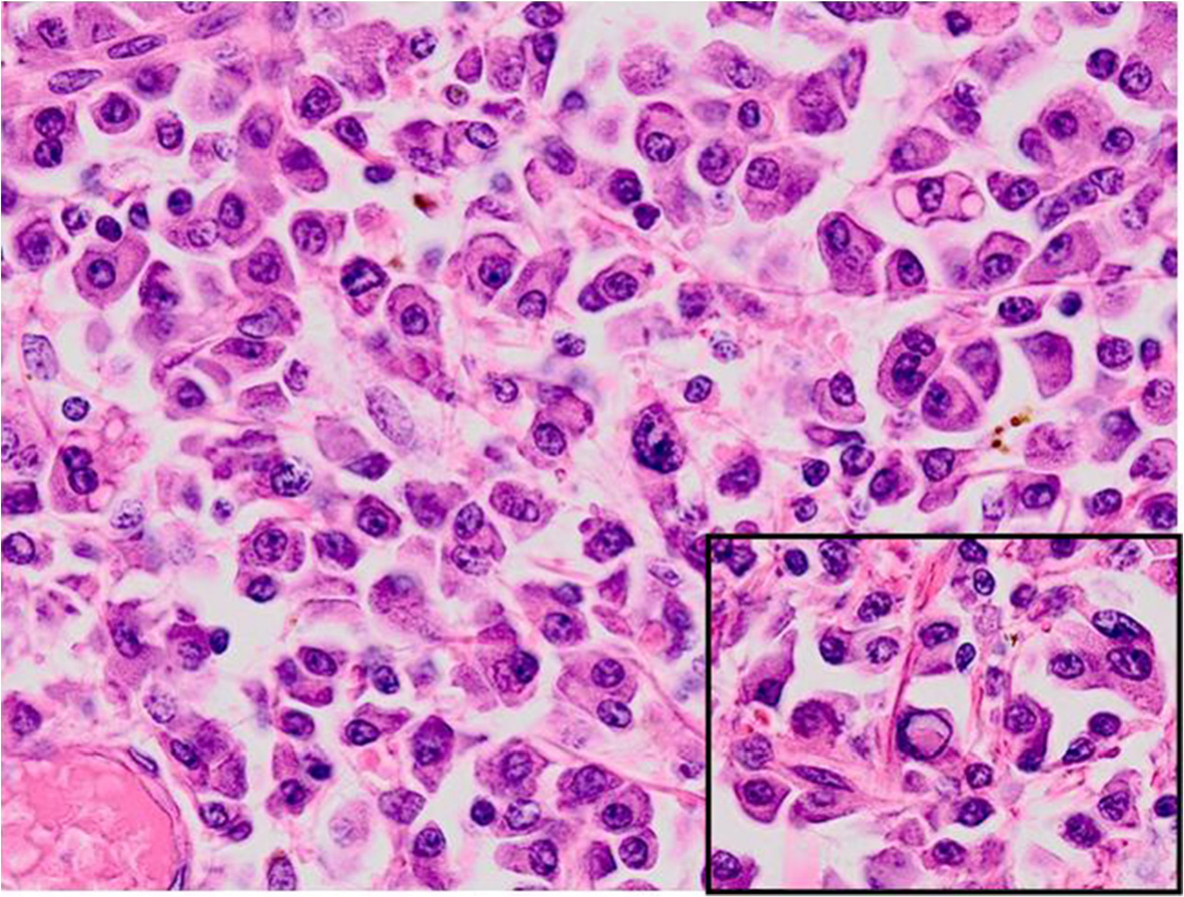
**The tumor consists of diffuse proliferation of mildly pleomorphic plasma cells.** Inset shows Dutcher bodies (HE stain).

**Figure 4:**
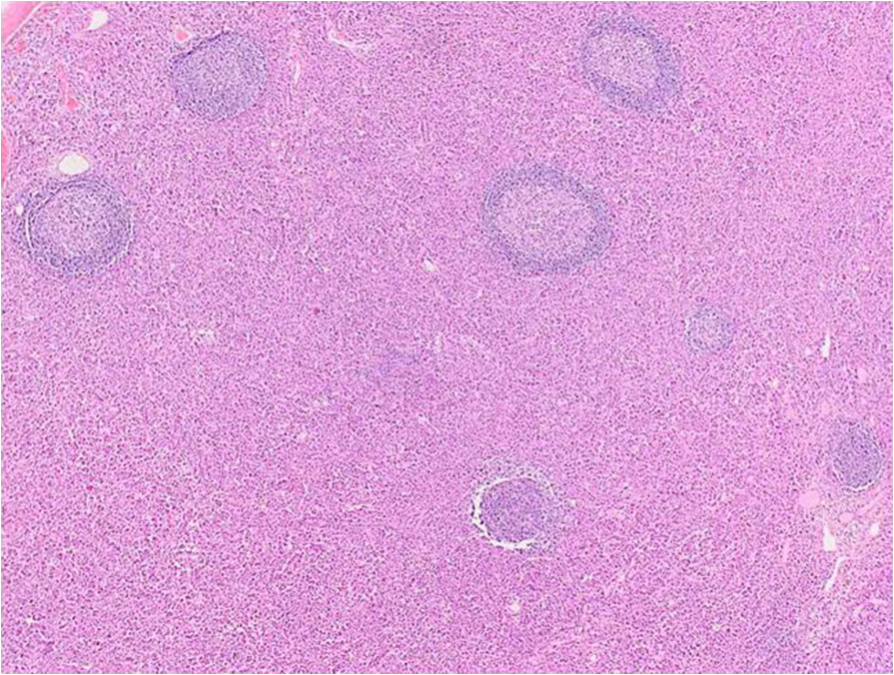
Numerous lymphoid follicles lay between the tumor cells (HE stain).

**Figure 5:**
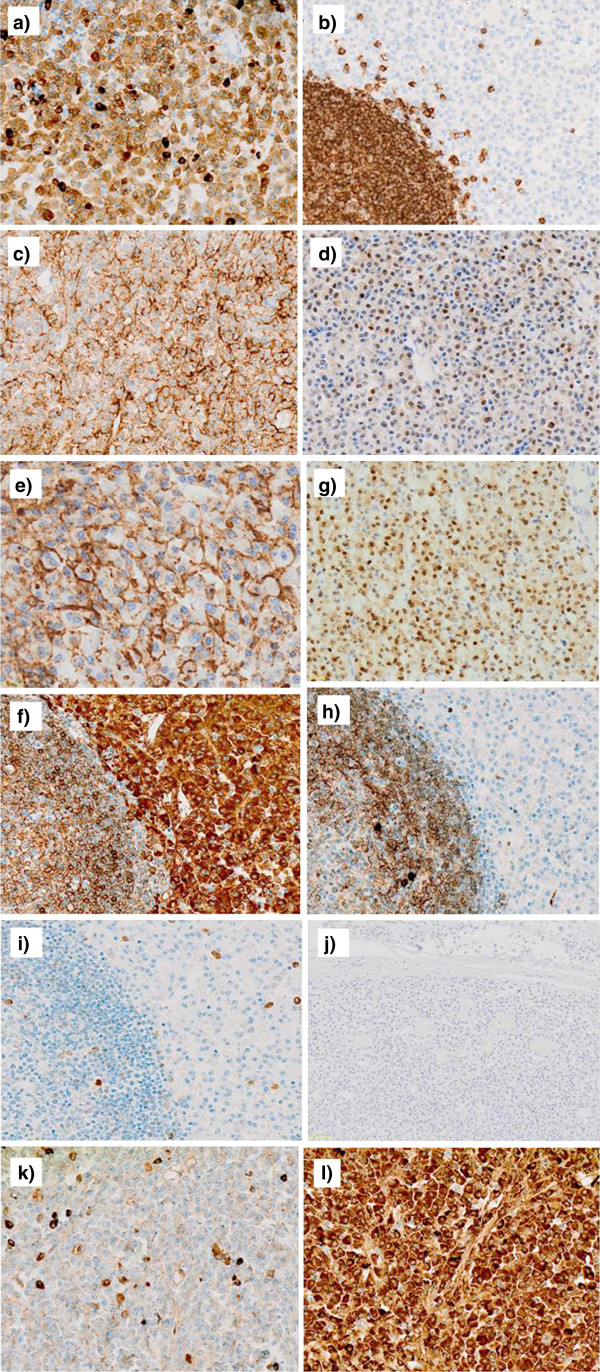
**Immunohistochemical studies. a**) CD79a is diffusely positive. The cells demonstrating strong positivity are reactive B-cells. **b**) CD20 is only positive in non-neoplastic B-cells in the scattered follicles, and in small amounts in interfollicular reactive cells. **c**) Bob-1, **d**) EMA, **e**) CD138 and **f**) MUM-1 are diffusely positive. Tumor cells are positive for **g**) IgA, and negative for **h**) IgG and **i**) IgM. **j**) D2-40 staining revealed no sinus-like structures in the tumor nodule. **k**) Tumor cell composite depicts absence of κ light chain expression, and **l**) diffuse cytoplasmic λ light chain expression.

Histologically, the tumor was relatively well-demarcated and entirely located in the submucosal layer (Figure 2). The surface mucosa covering the tumor was intact and was not associated with the tumor nodule itself, which consisted of a diffuse monomorphic proliferation of plasma cells (Figure 3). The nuclei of the tumor cells were peripherally displaced and mildly pleomorphic. Few tumor cells contained Dutcher bodies in their nuclei. Many lymphoid follicles were scattered throughout the tumor nodule (Figure 4) and featured well-formed mantle zones and normal looking follicular centers, which consisted primarily of large centroblasts with scattered tingible body macrophages. Giemsa staining showed no Hp adhering to the surface of the gastric mucosa. The bone marrow biopsy specimen was normal and offered no evidence suggestive of plasma cells proliferation. Immunohistochemical findings are provided in Figure 5. The tumor cells were diffusely positive for CD79a, Bob1, EMA, CD138, MUM-1, and IgA and negative for CD5, IgM and IgG. Although the lymphoid follicles and a small number of scattered reactive B-cells in the interfollicular zone were positive for CD20, the tumor cells were negative for CD20, CD19 and CD56. The immunophenotype of the germinal centers of the scattered lymphoid follicles was normal: CD10 positive, bcl-2 negative and preserved polarity in Ki67 (data not shown). *In situ* hybridization for κ and λ immunoglobulin light chains revealed λ light-chain restriction in the tumor cells. Additionally, D2-40 staining revealed no sinus-like structures in the tumor nodule, indicating that the nodule differs from the lymph node.

Given the laboratory results listed above, the lesions were diagnosed as primary submucosal nodular plasmacytoma of the stomach.

## Discussion

In this report we describe an extremely rare case of a primary nodular plasmacytoma developing in the gastric submucosa, which most likely constitutes a poorly recognized entity of primary gastric lymphoma.

With regard to neoplastic proliferation of plasma cells in the stomach, primary plasmacytoma should always be distinguished from other lymphoproliferative disorders with plasmacytic differentiation, such as plasma cell myeloma, lymphoplasmacytic lymphoma (LPL), plasma cell variant Castleman’s disease (PC-CD), and MALT lymphoma.

The systemic examination warrants bone marrow aspiration or biopsy in order to distinguish this condition from gastric involvement of osseous plasma cell myeloma. In the present case, no symptoms or findings suggestive of plasma cell myeloma were documented. In terms of immunohistochemistry, 67–79% of osseous plasma cell myeloma cases demonstrate CD56 positivity [7]. Although the results are ultimately inconclusive due to the limited sample number, CD56 negativity could perhaps be exploited as a tool for differentiating this disease from plasma cell myeloma.

The stomach is a relatively common site for extranodal lymphomas. While T-cell and NK/T-cell lymphomas are also known to sporadically affect the stomach, the majority of primary gastric lymphomas are of B-cell lineage [1,8]. MALT lymphomas most commonly arise in the GI tract and morphologically consist of heterogeneous small B cells, monocytoid cells, small lymphocytes, as well as scattered immunoblasts and centroblast-like cells [9]. Typically, neoplastic MALT lymphoma cells express markers of B-cell lineage, and are negative for cyclin D1, CD23, CD10 and CD5, with rare exceptions [10]. Plasmacytic differentiation is frequently present in gastric MALT lymphomas; however, they develop in the mucosa and are closely associated with involvement of the glandular epithelium. Thus they are frequently referred to as lymphoepithelial lesions. In addition, the majority of cases (62–77%) are associated with Hp infection [9]. The case presented here, however, revealed a completely distinct pattern of disease: well-demarcated “submucosal” nodules, a purely plasmacytic population of tumor cells, and no underlying Hp infection.

PC-CD is usually symptomatic, featuring fever, night sweats, weight loss and multiple lesions. PC-CD of the GI tract is extremely rare and there has been only one case report documenting a polypoid lesion 4 cm in diameter involving the rectum [11]. Typically, PC-CD shows marked proliferation of vessels in addition to prominent plasmacytic infiltration in the interfollicular areas. The lymphoid follicles show a reactive pattern with infrequent hyaline vascular features and penetrated vessels. In addition, PC-CD usually presents with a wide range of B cells and plasma cells are usually polyclonal; however, there have been reports detailing immunophenotypic evidence of Ig heavy and light chain restriction as well [12]. Therefore, a methodical morphological examination of the follicles and interfollicular areas is imperative to distinguish PC-CD from plasmacytoma. In the present case, none of the distinctive characteristics associated with PC-CD were identified.

LPL usually involves the bone marrow and the majority of patients possess an IgM serum paraprotein. Histologically, there is a relatively monotonous proliferation of small lymphocytes, plasma cells and plasmacytoid lymphocytes with relatively few transformed cells. Dutcher bodies, increased mast cells and hemosiderin are also typical features, and the κ light chain is expressed more frequently than the λ light chain. There is one case report on LPL occurring in the stomach; however, this was eventually disregarded as a gastric lesion of secondary involvement [13]. In the present case, the patient presented with no symptoms, and displayed no lymphadenopathy or serum paraproteins. In addition, the nuclei of the tumor cells were more pleomorphic and the non-plasmacytic lymphoid cells lacked a neoplastic component.

Usually MALT lymphomas or LPLs express IgM, while plasmacytomas express IgG. In the case reported here, the tumor cells were diffusely positive for IgA. Although plasmacytoma expressing IgA is a rare phenomenon, Shao et al recently reported the occurrence of a plasmacytoma expressing IgA, which featured young age at presentation, low risk of progression to plasma cell myeloma, and association with autoimmune disease or immune dysfunction [14]. In contrast, in our case, the patient did not present with any of the clinical features described by Shao et al.

Many previously reported cases of gastric plasmacytoma were essentially mucosal diseases [4-6] while our case showed a completely different pattern of proliferation. We therefore consider it to be a poorly recognized and rare variant of gastric plasmacytoma. To date, only two cases have reported gastric plasmacytomas located entirely in the submucosa as we describe in the present case [15,16]. A comparison of those cases and ours is shown in Table 1. The mean age of the three cases, consisting of two males and one female, was 61 years (range: 57–67 years). The mean tumor size was 2.1 cm (range: 0.7–4.1 cm). Resections were performed and none of the cases showed any evidence of tumor recurrence or development of systemic myeloma after the first treatment.Although a greater body of cases is needed to reach any definite conclusions, the experience with these three cases suggests that aggressive therapy is not required for patients who have undergone complete surgical excision. MALT lymphomas, along with certain cases of gastric plasmacytomas associated with Hp infection have reportedly been cured with Hp eradication [17,18]. However, no Hp infection was detected in our case or any of the other cases previously reported. Therefore, Hp eradication is presumably not an effective means of treatment for this subtype.

**Table 1:** Summary of gastric submucosal plasmacytoma

	**Age**	**Sex**	**Region in the stomach**	**Size(cm)**	**Therapy**	**Disease-free survival(year)**
Roost. et al(2007)	67	male	Angle	0.7	EMR*	2.5
Navin. et al(2010)	57	male	greater curvature	4.1	gastrectomy	no information
Our case	59	female	Corpus	1.5	Partial resection	1.5

*EMR, endoscopic mucosal resection.

Scattered ‘reactive’ lymphoid follicles in the tumor nodule are the defining characteristic findings in the present case. Although the two previously reported submucosal plasmacytoma cases were also well-circumscribed, the tumor described in this study presented with a pure consistency of plasma cells without the formation of lymphoid follicles. Although the exact reason as to why this tumor presented with scattererd lymphoid follicles is unclear, an underlying infection or nodal involvement are some plausible explanations. It is known that some infectious agents including Hp or Epstein-Barr virus induce inflammatory change with lymphoid follicles in the stomach [19,20]; however, Hp and in-situ hybridization for Epstein-Bar virus-encoded RNA(EBER) were both negative in this case. In addition, D2-40 immunostaining failed to reveal any well-developed sinus-like structures within the tumor nodule. It should also be noted that lymph nodes should not be present in normal submucosa. From these findings, it can be deduced that the possibility of nodal involvement is unlikely. However, the possibility of nodal involvement cannot be ruled out due to the presence of thin peripheral capsule-like structures, the well-demarcated morphology, and a few D2-40-positive vessels within the nodule. Further accumulation of similar cases and their subsequent analyses are required to clarify the full extent of these unique morphologies.

## Conclusions

To summarize, this report describes an extremely rare case of a primary solitary plasmacytoma situated exclusively in the gastric submucosa. This proliferation pattern is poorly recognized and most likely constitutes a novel variant of gastric lymphoma. Although the prognosis appears to be excellent, a greater number of similar cases is required in order to clarify the clinicopathological characteristics of this variant.

### Consent

Written informed consent was obtained from patient for publication of this case report and accompanying images. A copy of the written consent is available for review by the Editor-in-Chief of Diagnostic pathology.

## Competing interests

We do not have any competing interests for our manuscript.

## Authors’ contribution

MK was responsible for data collection and drafted the manuscript. TI helped to revise this manuscript. CH, YK, FK, SH helped to diagnosis. HM was the patient’s attending physician and made contribution to acquision of clinical data. All authors read and approved the final manuscript.
